# Outcome after Discontinuing Long-Term Benzimidazole Treatment in 11 Patients with Non-resectable Alveolar Echinococcosis with Negative FDG-PET/CT and Anti-EmII/3-10 Serology

**DOI:** 10.1371/journal.pntd.0003964

**Published:** 2015-09-21

**Authors:** Rudolf W. Ammann, Katrin D. M. Stumpe, Felix Grimm, Peter Deplazes, Sabine Huber, Kaja Bertogg, Dorothee R. Fischer, Beat Müllhaupt

**Affiliations:** 1 Department of Gastroenterology and Hepatology, University Hospital of Zurich, Zürich, Switzerland; 2 Institute of Radiology, Hirslanden Hospital, Zürich, Switzerland; 3 Institute of Parasitology, University of Zurich, Zurich, Switzerland; 4 Division of Nuclear Medicine, University Hospital Zurich, Zürich, Switzerland; 5 Swiss HPB (Hepato-Pancreato-Biliary) Center, University Hospital of Zurich, Zürich, Switzerland; Universidad Peruana Cayetano Heredia, LIMA

## Abstract

**Background/Aims:**

Benzimidazoles are efficacious for treating non-resectable alveolar echinococcosis (AE), but their long-term parasitocidal (curative) effect is disputed. In this study, we prospectively analyzed the potential parasitocidal effect of benzimidazoles and whether normalization of FDG-PET/CT scans and anti-Emll/3-10-antibody levels could act as reliable "in vivo" parameters of AE-inactivation permitting to abrogate chemotherapy with a low risk for AE-recurrence.

**Method:**

This prospective study included 34 patients with non-resectable AE subdivided into group A (n = 11), followed-up after diagnosis and begin of chemotherapy at months 6, 12 and 24, and group B (n = 23) with a medium duration of chemotherapy of 10 (range 2–25) years. All patients were assessed by FDG-PET/CT examinations and anti-EmII/3-10 serology. Chemotherapy was abrogated in patients with normalization of FDG-PET/CT and serum anti-EmII/3-10 levels. These patients were closely followed-up for AE recurrence. Endpoint (parasitocidal efficacy) was defined by the absence of AE-recurrence >24 months after stopping treatment.

**Results:**

Normalization of FDG-PET/CT scan and anti-EmII/3-10 levels occurred in 11 of 34 patients (32%). After abrogation of chemotherapy in these 11 patients, there was no evidence of AE-recurrence within a median of 70.5 (range 16–82) months. However, the patients’ immunocompetence appears pivotal for the described long-term parasitocidal effect of benzimidazoles.

**Conclusions:**

The combination of negative FDG-PET/CT-scans and anti-EmII/3-10 antibody levels seem to be reliable parameters for assessing in vivo AE-larval inactivity after long-term benzimidazole chemotherapy.

**Trial Registration:**

clinicaltrials.gov: NCT00658294

## Author Summary

Alveolar echinococcosis is one of the mostly deadly human parasitic diseases if left untreated. The treatment of choice is surgical resection, followed by two years of benzimidazole treatment. Unfortunately, only about 30–40% of patients have a resectable disease, while the others require medical treatment with benzimidazoles. As this therapy is only considered to be parasitostatic and as there are not yet reliable tools to assess parasite viability, the treatment usually is life-long. In this study, we evaluated FDG-PET/CT and antibody levels against the recombinant Emll/3-10 antigen as markers for parasite viability, allowing to select patients in whom chemotherapy could be stopped with low risk of AE-recurrence. Eleven 11 patients were identified with negative FDG-PET/CT-scans and anti-EmII/3-10 antibody levels in whom benzimidazole treatment was stopped with no evidence of AE-recurrence within a median follow-up of 70.5 (range 16–82) months. Therefore, this study provides evidence that benzimidazole treatment is parasitocidal in a subset of patients.

## Introduction

Alveolar echinococcosis (AE), one of the most Iethal human helminthic infections, is acquired by ingestion of eggs from *Echinococcus multilocularis*. AE behaves biologically like a malignant tumor with a tendency to metastasize into distant organs [1–5]. The treatment of choice is radical resection, however, only 30–40% of patients have resectable liver lesions [1,4,6,7]. In the pre-chemotherapy area, the 10-year survival rate of patients with non-resectable AE was less than 10% [1,8]. With the advent of benzimidazole based chemotherapy, the outcome of non-resectable AE has dramatically improved. Long-term chemotherapy with benzimidazoles improves the 10-year survival rate of non-resectable AE to 80% [1,8–10]. However, whether long-term chemotherapy is parasitocidal (curative) or remains parasitostatic, with the need for life-long therapy is still unclear [11–15]. The lack of reliable non-invasive methods for assessing parasite viability remains a major problem. Thus, absence of AE-recurrence two to three years after stopping chemotherapy is retrospectively considered as evidence of a curative effect [11,12,14].

Recently, FDG-PET (fluorodeoxy-glucose-positron emission tomography) [16,17] and antibody levels against the recombinant EmII/3-10 antigen [18] have been described as two new promising methods for assessing parasite viability in patients.

Increased FDG-uptake in functional PET-imaging is observed in tumoral, inflammatory and infectious lesions. Decrease in FDG-uptake is considered as a useful metabolic parameter for assessing anti-tumoral/anti-infectious pharmacotherapy [19–22]. It was hypothesized that an increased FDG-uptake of AE lesions reflects parasite viability [16,17]. The reverse assumption that a decrease in FDG-uptake during chemotherapy may be a reliable parameter of parasite death, however, was not confirmed in a recent study [14].

Serum levels of specific anti-Emll/3-10 antigen appear to be another promising parameter for parasite viability [18]. In a preliminary study, a putative parasitocidal effect was observed in two third of patients with non-resectable AE following abrogation of long-term chemotherapy [12]. A recent follow-up study showed that the anti-Emll/3-10 profile normalized in 8 of 9 patients without AE-recurrence. In contrast, high antibody levels were found in 7 of 9 patients with AE-recurrence [18].

The aim of the present study was to prospectively evaluate whether long-term chemotherapy is parasitocidal in patients with AE, and whether FDG-PET/CT in combination with serum anti-Emll/3-10 levels can be used to select patients in whom chemotherapy can be stopped with a low risk of AE-recurrence. This is clinically highly relevant because the frequency of AE is rising in Europe and Asia [10,23–25], and the parasite is emerging in parts of North America [26] and furthermore the costs associated with long-term chemotherapy are high (up to 16 300 Euro/patient-year) [9].

## Methods

### Study design

Prospective treatment abrogation study including patients with non-resectable AE and previous chemotherapy with either albendazole or mebendazole for at least two years. The benzimidazole dose was adjusted to reach appropriate serum level 4 hours after the morning dose (albendazole: >1umol/l, mebendazole:>250 nmol/l) [7].

Inclusion criteria: Non-resectable AE, benzimidazole treatment for at least two years, absence of FDG uptake (negative FDG-PET/CT scan) and negative anti-EmII/3-10 levels.

Exclusion criteria: Positive FDG-PET/CT scan and/or detectable anti-EmII/3-10 levels, non-Compliance of the patient, reduced life expectation (age >80 years, concomitant malignoma, preterminal disease e.g. renal, hepatic, or pulmonal) and pregnancy.

Patients were selected among a group 34 patients with non resectable AE lesions. Subgroup A included 11 patients with newly diagnosed AE who had been closely followed by FDG-PET/CT (at 6, 12 and 24 months) and EmII/3-10 serology during the initial years of chemotherapy [17]. Subgroup B comprised 23 patients under long term benzimidazole therapy (up to 25 years), who were either inoperable (n = 15) or had either a recurrence after resection (n = 3) or an R1 resection (n = 5). They were monitored regularly by EmII/3-10 serology (every 6–12 months) and imaging. At variable time intervals after starting albendazole treatment a FDG-PET/CT was obtained.

Patients form both groups that fulfilled the inclusion criteria were included in the study and stopped treatment. After treatment stop all patients were followed prospectively with Emll/3-10 serology every 3 months. Imaging studies were performed at start and then every 12 months or at shorter intervals when AE-recurrence was suspected.

### Ethics statement

The study was performed according to the declaration of Helsinki. The protocol was approved by the ethical committee of the canton Zurich (Kantonale Ethikkommission des Kanton Zürich), Switzerland and written informed consent was obtained from all patients (clinicaltrials.gov:NCT00658294).

### Study procedures

The FDG-PET/CT protocol has recently been published [21]. Images were analyzed by 2 board certified nuclear physicians as described previously and a semi-quantitative FDG-uptake grading scale from 0 to 4 was applied [21].

ELISA using the recombinant Emll/3-10 antigen was performed as previously reported [18,27]. Antibody levels < 5 AU (arbitrary antibody units) were considered negative.

### *Echinococcus multilocularis* PCR

*Echinococcus multilocularis*-DNA was detected by PCR in fresh tissue samples obtained at surgery using primers H15 and H17 that amplify a *E*. *multilocularis* specific fragment of the mitochondrial 12S rRNA gene [28].

### Disease classification

The disease stage was classified using the PNM system (S1 Table) [29].

### Follow-up

Follow-up (FU) is defined as the time from diagnosis to the last contact, the cut-off date (December 2012) or the death of the patient.

AE-recurrence was defined if new lesions were detected and/or by progression of lesions on imaging in association with a positive Emll/3-10 serology and/or a positive PET-CT. A positive PET-CT alone was not sufficient to diagnose AE-recurrence.

### Statistics

Differences between groups were analysed with Chi Square test or Fisher’s exact test using SPSS software, version 20 (IBM Corporation Armonk, New York, United States). The 95% Confidence Interval (95% CI) was calculated using RStudio, Version 0.98.1062, 2009–2013 RStudio, Inc.

## Results

The baseline demographic of **group A patients** (n = 11) are summarized in Table 1. Nine patients had an advanced PNM stage (lllb or IV). Two patients with less advanced AE-Iesions (stage I to lIIa) were treated by long-term chemotherapy because of serious comorbidity (A1, A6).

**Table 1:** Typical findings of 11 newly diagnosed non-resectable AE-patients at baseline and at 2 years of initial chemotherapy. Group A

Nr	Sex	Age at diagnosis	PNM at diagnosis	PET grade (0–4)		Anti-EmII/3-10 (>5 AU)		Cessation of therapy	Comment
				at baseline	at 2 yrs.	at baseline	at 2 yrs.		
A1	M	39	P3N0M0	2	2	+	+	no	HIV
A2	F	52	P4N0M0	4	2	+	+	no	
A4	F	41	P4N0M0	4	4	+	+	no	
A6	F	72	P1N0M0	3	4	+	neg.	no	Immuno-suppression
A10	M	61	P4N1M0	3	4	+	+	no	
A11	F	55	P4N1M0	3	4	+	+	no	
A3	M	60	P4N0M0	3	0	+	neg.	yes	
A5*	F	52	PXN0M0	2	0	neg.	neg.	yes	
A7	F	62	P4NIM0	4	0	+	neg.	yes	
A8	F	60	P4N1M0	0	0	neg.	neg.	yes	
A9	M	64	P3N1M0	4	0	+	neg.	yes	

Median age (range), yrs.: 60 (39–72)

*Recurrence after presumed radical surgery

One patient presented with a negative EmII/3-10 serology and PET-negative lesions (A8) and another one had a negative EmII/3-10 serology at baseline (A5). Nevertheless, both patients got regular chemotherapy for safety/ study protocol reasons for a minimum of 2 years.

Overall, 5 out of 11 patients of group A qualified for abrogation of chemotherapy and were included into the study (42%; 95% CI: 16.7–76,6%) (A3, A5, A7, A8, A9).

The pertinent characteristics of **group B patients** (n = 23) are summarized in Table 2. The 23 patients had been treated for up to 25 years by chemotherapy (median 10, range 2–25 years). AE-recurrence following "radical" surgery was observed in 3 patients (B1, B2, B6) and 5 additional patients had undergone R1 surgical resection many years ago (B3, B5, B13, B19, B21 (Table 2). Furthermore, 4 of the 23 patients had AE-recurrence following abrogation of chemotherapy in our previous study (B1, B2, B6, B16) [12]. In group B, 7 out of 23 patients qualified for abrogation of chemotherapy (30%; 95% CI: 13.2–52.9%). Chemotherapy was not stopped in one of them because of metastatic breast cancer with unclear prognosis (B19).

**Table 2:** Typical findings of 23 patients with non-resectable AE treated by chemotherapy for up to 25 years. Group B

Nr	Sex	Age at diagnosis	Treatment duration (yrs.) before FDG PET-CT	PNM	PET grade (0–4)	Anti- EmII/3-10 (>5)	Cessation of therapy
B1*	M	43	22	P3N1M1	0	+	no
B2*	F	33	24	P3N1M0	3	normal	no
B3**	F	29	21	P3N1M0	3	+	no
B4	F	63	10	P3N1M0	3	normal	no
B5**	F	51	8	P3N1M0	3	normal	no
B6*	M	34	22	P4N1M0	0	+	no
B7	F	29	14	P3N1M0	3	normal	no
B8	F	17	8	P4N0M0	3	+	no
B9	F	72	16	P2N1M0	3	normal	no
B10	F	59	23	P3N1Mo	3	+	no
B11	M	44	4	P3N0M0	3	+	no
B12	F	66	4	P3N1M0	3	+	no
B19**	F	44	25	P3N1M0	0	normal	no
B20	M	85	2	P4N1M0	3	normal	no
B21**	M	48	3	P4N1M0	3	+	no
B22	M	44	2	P4N1M0	3	normal	no
B23	M	37	3	P4N1M0	3	normal	no
B13**	M	37	15	P3N0M0	0	normal	yes
B14	F	47	4	P3N1M0	0	normal	yes
B15	M	52	20	P3N1M0	0	normal	yes
B16	F	36	23	P3N0M0	0	normal	yes
B17	M	62	6	P1N1M0	0	normal	yes
B18	M	45	9	P3N1M1	0	normal	yes

Median Age (range), yrs.: 44.0 (17–85)

Median duration of chemotherapy (range), yrs.: 10 (2–25)

* Recurrence after presumed R0 surgery (RR)

** R1 resection

Patients in group B were significantly younger (median age: 44 yrs. compared to 60 yrs., p<0.001), but gender distribution and PNM-classification were similar.

**Fig 1:**
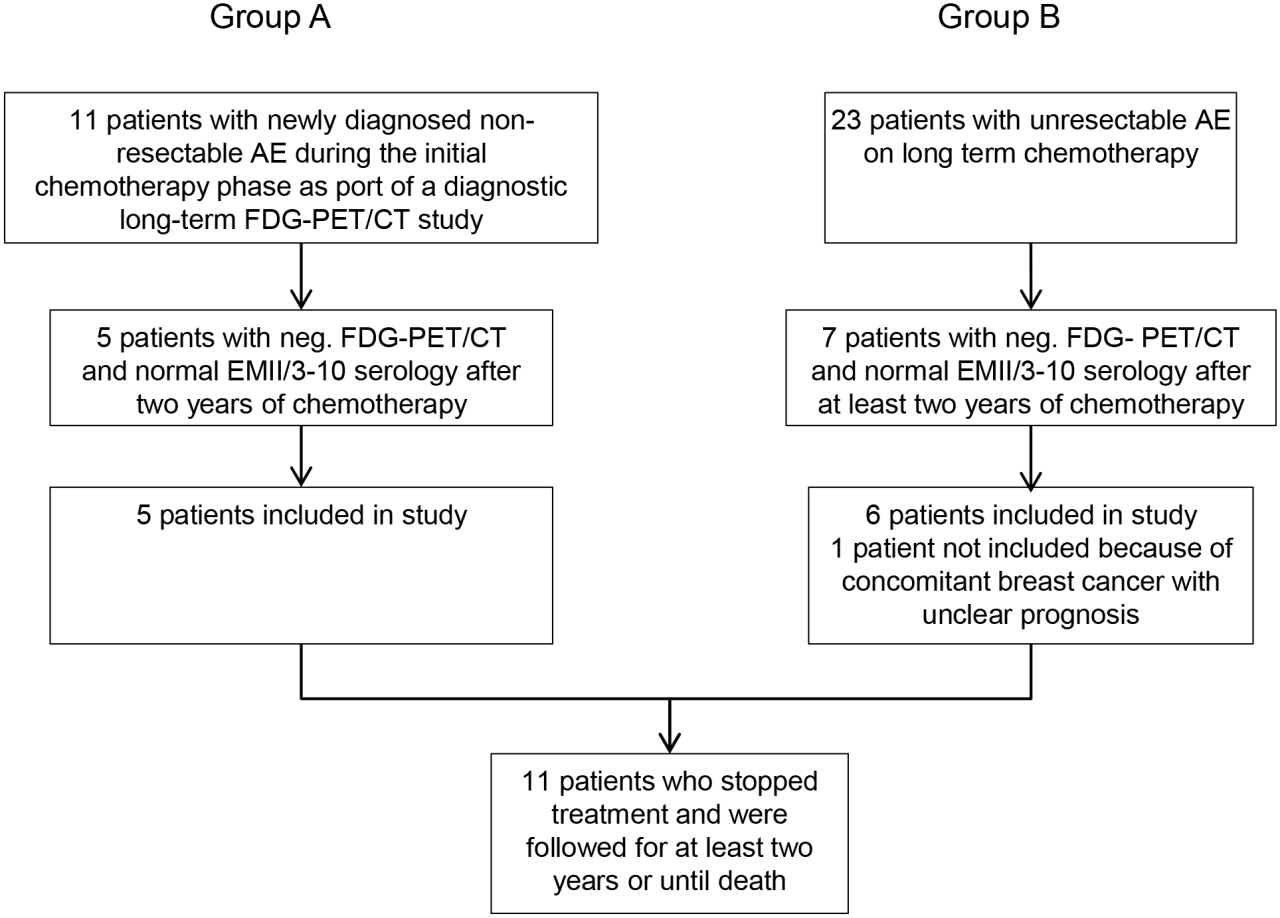
Disposition of the study population.

The course and the final assessment of the 11 patients of groups A and B that stopped chemotherapy, representing the final study population (Fig 1), are summarized in Table 3. The median follow-up after abrogation of chemotherapy was 70.5 (range 16–82) months. Patients who qualified for treatment abrogation were significantly older (median age 52 yrs. vs. 44 yrs.; p<0.001), but gender distribution as well as the number of advanced stages (stage IIIb-IV) between final study population (Table 3) and group A and B, respectively, (Tables 1 and 2) were similar.

**Table 3:** Follow-up (FU) after abrogation of chemotherapy in non-resectable AE.

Nr	Sex	Age at diagnosis (yrs.)	Treatment duration (mo) before treatment abrogation	Follow-up after stopping therapy (mo)	EmII/3-10 (>5)	Final assessment
A9	M	64	34	71	normal	No AE recurrence
A7	F	62	36	70	normal	No AE-recurrence: Short stricture of hepatic duct due sclerosing cholangitis. Successful endoscopic treatment.
A8	F	60	36	70	normal	New PET pos. lesion close to AE-scar at 48 months. Surgery: necro-granulomatous lesion. AE unlikely. No chemo-therapy. Follow-up two years later: scar
A3	M	60	37	16	normal	Died due to pancreatic cancer. No AE activity in resection specimen
B14	F	47	41	60	normal	37 mo. After stopping chemotherapy new cystic, PET negative lesion close to AE-scar. AE recurrence unlikely. PET neg scar after two additional year of follow-up
A5	F	52	50	68	normal	No AE recurrence
						Choledocholithiasis
B17	M	62	78	82	normal	No AE recurrence
B18	M	45	108	75	normal	No AE recurrence
B13	M	37	180	56	normal	No AE recurrence
B15	M	52	240	78	normal	No AE recurrence
B16	F	36	276	76	normal	No AE recurrence

Median age (range), yrs.: 52 (36–64)

Median duration of treatment (range), mo.: 50 (34–276)

Follow-up after stopping treatment (range), mo.: 70 (16–82)

The clinical course following cessation of chemotherapy was uneventful without any evidence of AE-recurrence in 6 patients. In 5 patients however, the course after cessation of chemotherapy was complicated by incidental findings (Table 3).

**Fig 2:**
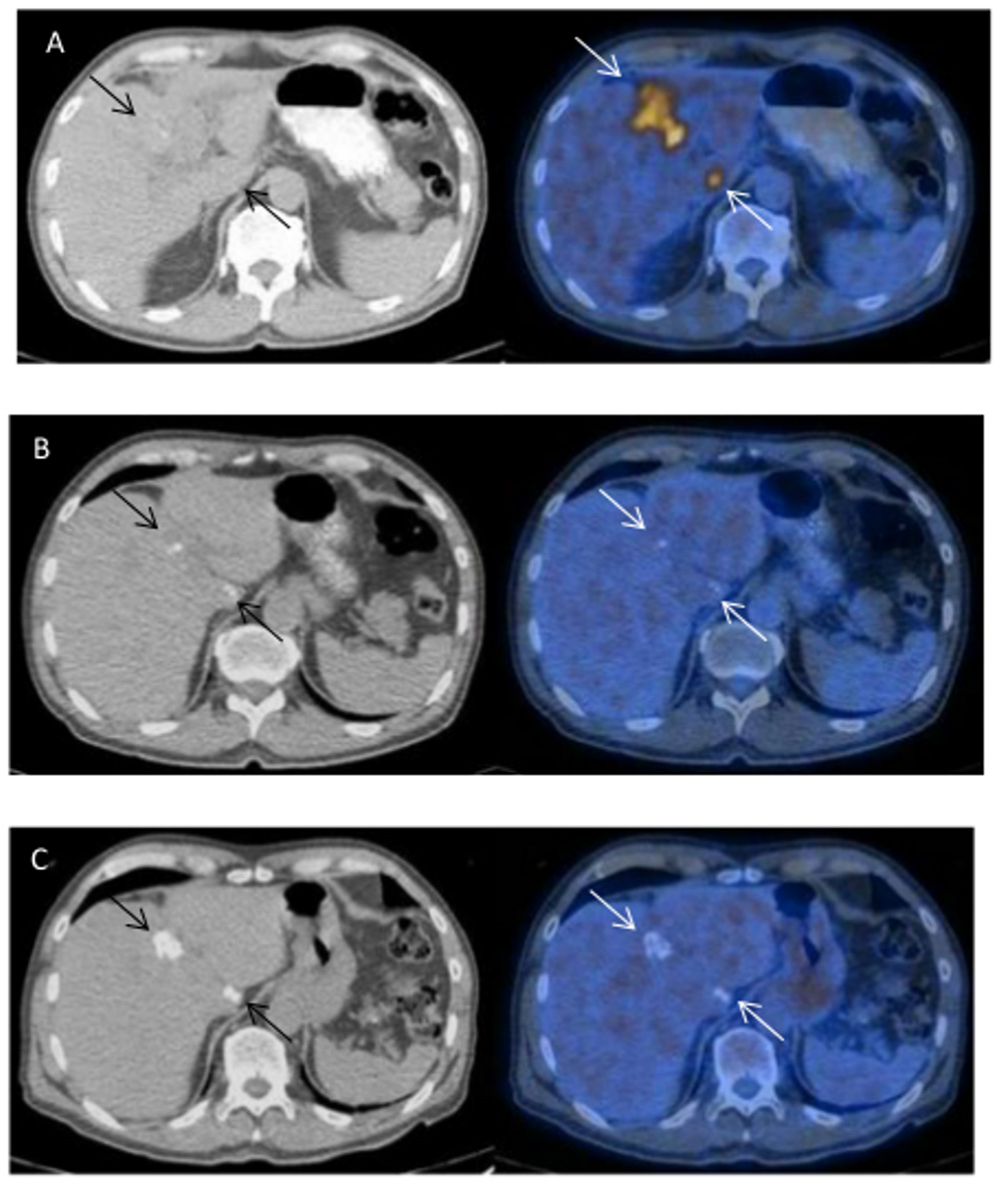
(A9): Baseline and follow-up FDG-PET/CT scans without i.v. contrast. a) Large and small AE-lesions (arrow) at the time of diagnosis (2003) with very strongly increased FDG uptake. b) The lesions became FDG-negative within two years of albendazole treatment. c) Progressive calcifications of AE lesions over the next 6 years after abrogation of chemotherapy (2011). No signs of FDG uptake are present.

Overall, benzimidazole therapy was probably parasitocidal in at least 32% (11/34; 95% CI 17.4–50.5%) of all patients with non-resectable AE since recurrence was not observed after a median follow-up of 70.5 (range: 16–82) months following abrogation of chemotherapy (0%, 0/11; 95% CI: 0–28.5%). There was no correlation between the initial size of the FDG-PET/CT positive lesion and the chemotherapeutic efficacy (shown for patient A9 in whom therapy was parasitocidal despite an extensive initial lesion (PNM stage P3N1M0; Fig 2)).

Interestingly, anti-Emll/3-10 antibody levels were very high in 5 of 6 subgroup A patients (126.5 ± 58.6 AU) who did not fulfill the inclusion criteria and in whom no parasitocidal effect could be noted (A1-2, A4, A6, A10-11). In contrast, the 5 patients in whom a parasitocidal effect was obvious had substantially lower anti-EmII/3-10 antibody values (30.5 ± 42.9 AU) (A3, A5, A7-9)(Table 4).

**Table 4:** Long-term follow-up of anti-EmII/3-10 levels in patients of group A.

	Baseline	6 months	12 months	24 months	36 months	48 months	60 months	72 months	84 months	96 months
A1	132	92	57		12	7	0	5	11	7
A2	172	151	133		84		98	109	150	
A3	41	19	0	0	0	0	0			
A4	145	128	119	128		183	253	262	212	103
A5	0	0	0	0	0	0	0	0	0	0
A6	11	0	0	0		0	0	0		
A7	110	75	72	0	0	0	0	0	0	0
A8	0	0	0	0	0	0	0	0	0	0
A9	32	0		0	0	0	0	0	0	0
A10	133	103	112			53	50	10	39	24
A11	166	144	127	124		160	276	265	238	

### Complicated clinical courses

One patient (**A3**) underwent a Whipple operation for pancreatic cancer 4 months after abrogation of albendazole treatment. At surgery, the hepatic AE-Iesion was resected and no viable AE-tissue was histologically found. The patient died from metastatic pancreatic cancer 16 months after stopping chemotherapy. No autopsy was performed.

**Fig 3:**
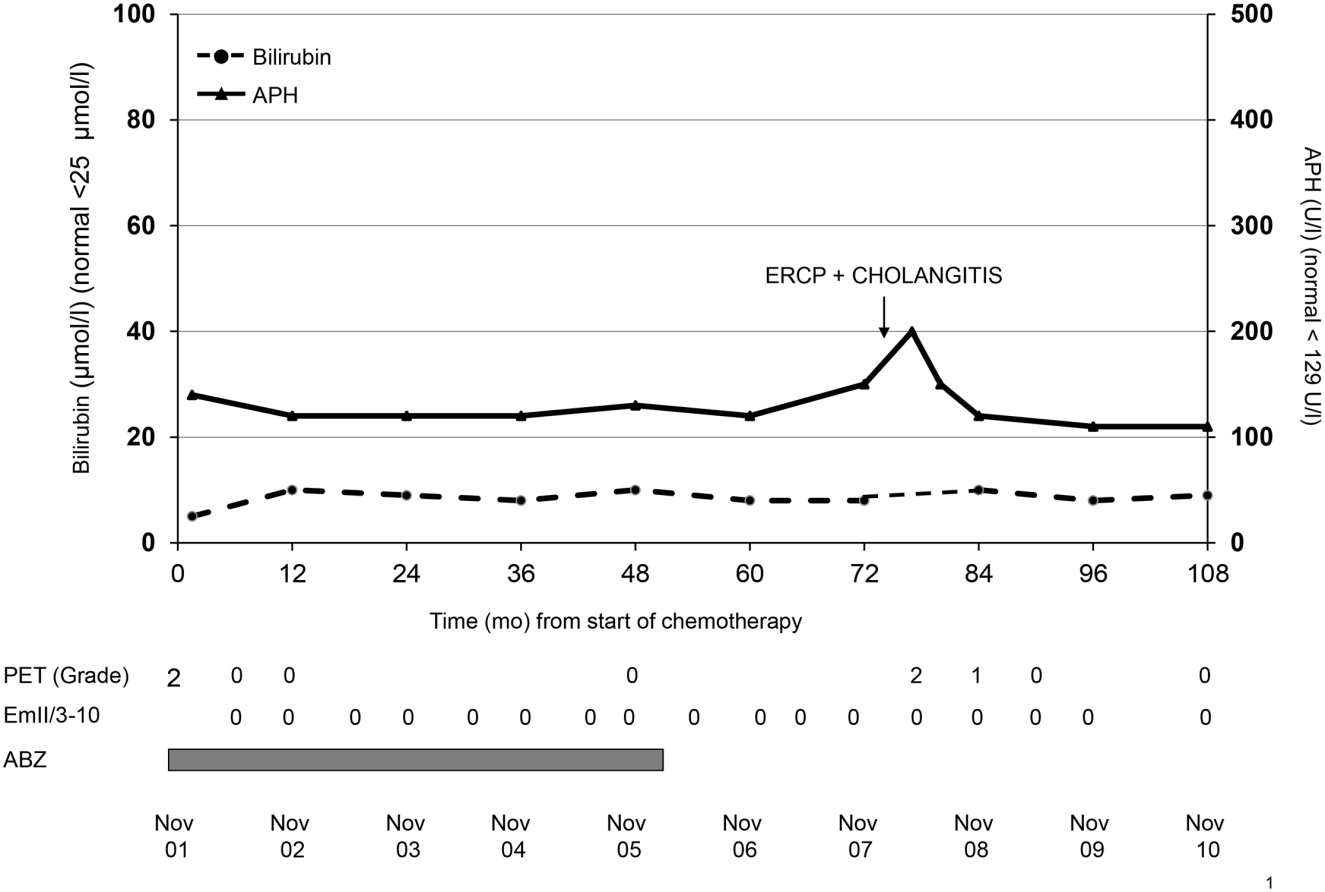
(A5): Long-term follow-up of a large AE recurrence (Sept 2001), 3 years following presumed radical surgery). Albendazole was administered from Sept. 2001 until February 2006. Oligosymptomatic choledocholithiasis was treated by ERCP with papillotomy and stone extraction in April 2008. The procedure was complicated by cholangitis, which was treated by long-term antibiotic therapy. No AE-recurrence was noted during 70 months of follow-up after stopping albendazole treatment.

The second patient (**A5**) underwent a presumed radical hemihepatectomy in 1988 for AE followed by insufficient mebendazole treatment for only 3 months (Fig 3). In September 2001 a severe AE-recurrence with a large lesion (7 x 12.5 cm) was detected, which was FDG-PET/CT positive and showed the typical radiological findings of AE, but anti-Emll/3-10 antibody levels remained negative. There was a rapid response to chemotherapy (Sept 2001 to Feb 2006). Albendazole was abrogated in February 2006 despite mild cholestasis that persisted since Dec 2001. In April 2008, endoscopic sphincterotomy with stone extraction was performed. The procedure was complicated by cholangitis and a perihepatic fluid collection, showing strongly increased FDG-uptake in FDG-PET/CT. Long-term antibiotic treatment was administered but no benzimidazole was given. Actually, cholestasis has regressed to almost normal values, FDG-PET/CT became negative again and anti EmII/3-10 levels remained negative all the time.

A third patient (**A7**) presented in 2001 with painless jaundice and a large (10 cm) necrotic AE-Iesion of the right lobe with extension to liver hilum (non-resectable). Response to albendazole treatment was excellent. FDG-PET/CT and EmII/3-10 serology became negative. Medical treatment was stopped in 2005 despite persistent mild cholestasis. Abrogation of chemotherapy was followed by marked painless jaundice. ERCP in June 2006 showed a filiform (2 cm) stricture of the common bile duct compatible with "sclerosing-cholangitis" as previously described [30]. FDG-PET/CT and anti-Emll/3-10 levels remained negative. The biliary obstruction was successfully treated by endoscopic intervention including stenting. Since AE recurrence could not be excluded and the patient developed at approximately the same time an inoperable uterine cancer, which was treated with irradiation and cytostatic therapy, albendazole treatment was resumed and continued until the last follow-up (Oct. 2012), even though a firm proof of AE recurrence was lacking.

In the next patient (**A8**), albendazole therapy was carried out from Oct 2002 to Nov 2005 for non-resectable AE (segment V, VI, VII). After treatment abrogation (Nov 05), the clinical course over the next 3 years was uneventful. In Dec. 2008, a markedly elevated sedimentation rate (clinically unexplained) was noted which persisted also in July 2009. In Oct. 2009, a new cystic lesion adjacent to the preexistent AE-Iesion (segment VI) was detected by CT. The anti-Emll/3-10-antibody levels were negative, and the lesion showed diffusely increased FDG-uptake in PET/CT. The lesion was resected and a sterile, subacute necro-granulomatous inflammation of unknown etiology was revealed by histology. *Echinococcus multilocularis* PCR results were negative. Therefore, an AE recurrence seemed highly unlikely. Three years, later at the last follow-up (November 2012), no evidence for AE recurrence was noted.

In patient B14, a non-resectable AE-lesion of the right and caudate liver lobe with partial occlusion of the portal vein was treated with albendazole between Jul. 2003 and Dec. 2009. The treatment was well tolerated, and the patient qualified for abrogation of therapy in Dec. 2009. However, a new cystic lesion was detected by CT in segment VI, adjacent to the previous AE-lesion. A wait-and-see strategy was adopted with regular follow-up but without resumption of chemotherapy. Follow-up was uneventful, and a CT scan showed a calcified scar, but no evidence of AE-recurrence in Nov. 2012.

## Discussion

Our prospective long-term study provides strong evidence that chemotherapy was parasitocidal in at least 11 out of 34 patients (32%) with non-resectable AE. These results contradict the current opinion of an exclusively parasitostatic effect with the need for lifelong medical therapy [2–4,7,9,14]. Moreover, patient follow-ups by FDG-PET/CT and Emll/3-10 serology represent a strategy to assess parasite viability and to decide whether treatment can be safely discontinued.

Our data seem to be in discordance with a clinical study including 23 patients with non-resectable AE in Ulm (Germany) [14]. Based on negative FDG-PET-scans, benzimidazole therapy of variable duration was stopped in 15 patients, but evidence of AE-recurrence was noted in 53% within the following 18 months. These data suggested a poor correlation between FDG-PET-scan results and larval viability [14].

In contrast to our study (33% negative FDG-PET/CT), the rate of FDG-PET negative lesions after long-term chemotherapy was surprisingly high (65%) in the Ulm study.

In previous studies, the interval between abrogation of chemotherapy and AE recurrence averaged 33.6 (range 12–156) months [12,18] and less than 18 months [14]. No AE-recurrence was noted on an average 29.2 (range 6.8–66) and 23 (range 8–37) months, respectively, after cessation of chemotherapy in two recent series from the same group (n = 5 and n = 7) [31,32].

In the present series, the patients were followed for a median of 70 (range 16–82) months after abrogation of chemotherapy. Accordingly, the risk of missing AE-recurrence after stopping chemotherapy in our series appears small, although AE-recurrence has been reported in single cases after 106 [33] and 156 months [8] respectively.

The problem of diagnosing AE-recurrence following abrogation of chemotherapy is illustrated by the numerous incidental findings in our series (A3, A5, A7, A8, B14). In particular, cholestasis after chemotherapy abrogation was probably not caused by AE-recurrence (A5, A7), since follow-up data were compatible with a sclerosing-cholangitis like syndrome, a largely unknown syndrome [30] in one and oligosymptomatic (incidental) choledocholithiasis in the second patient.

Two patients of our series developed new liver lesions (A8, B14). Causes are unexplained, but AE-recurrence seemed very unlikely particularly because levels of specific antibodies (including anti-Emll/3-10 antibodies) remained unchanged and no lesion progression was observed during a three year follow-up period without chemotherapy.

According to the current literature, the predictive value of post-chemotherapy immunsurveillance is limited [2,4,9,34,35]. In contrast, our data indicate that anti-EmII/3-10 levels are valuable for assessing parasite viability ([18], present series). In our experience, the anti-Emll/3-10 levels are more sensitive markers for larval viability than the serological results obtained by the Em2-plus test containing two antigens (Em2 and EmII/3-10) [36] used by Reuter et al. [14].

Surprisingly, we found that baseline anti-Emll/3-10 levels appear to have a predictive value with regard to a parasitocidal efficacy of albendazole treatment. These levels were very high (>120 AU units) in 5 of 6 patients of group A in whom no parasitocidal effect was observed, in contrast to the 5 patients with low levels (< 120 AU) in whom there was a probable parasitocidal effect (Fig 3). The predictive value of baseline anti-EmII/3-10 serology for parasitocidal vs. parasitostatic efficacy deserves further studies in larger series of AE patients.

It is largely unknown which factor(s) are important for the parasitocidal effect of benzimidazole treatment. It has been suggested that a parasitocidal effect may be related to the duration of therapy [11,15]. This assumption was not confirmed in recent studies [14] and the present series. In particular, no parasitocidal efficacy was noted in the majority of 23 patients (group B) despite chemotherapy for up to 25 years (Table 2).

The parasite host-immune-interaction probably plays an important role for the outcome of AE-infection [37]. First, AE-lesions may be inactivated spontaneously i.e. “died-out” AE [2,4,37,38]. Second, the impact of immune deficiency as (co)-factor for lacking parasitocidal efficacy is supported by 2 patients in group A, i.e. HIV-infection (A1) or continuous immunosuppressive therapy (rheumatoid arthritis; A6). According to a recent French series, a family clustering of AE was noted in 20 of 153 patients (13%) [39]. Such an association was not observed in our series. Third, AE progression in AIDS [40] or in immune-compromised patients following liver transplantation [41] as well as in animal experiments with cyclosporine-induced immunodeficiency [42] emphasize the impact of an intact immune system on the evolution of AE disease.

In conclusion, according to our knowledge, this is the largest prospective study documenting a parasitocidal efficacy of long-term benzimidazole chemotherapy in 11 patients with AE. Negative anti-EmII/3-10 levels combined with normalized FDG-PET scan were reliable parameters to predict a recurrence-free survival after stopping benzimidazole treatment. Until our data are confirmed long-term benzimidazole treatment is still the standard of care for patients with unresectable AE, after R1 resection or after liver transplantation [2]. To stop long-term benzimidazole treatment should only be considered in experienced center using the same approach as outlined in this manuscript. Especially positive PET-CT findings should be carefully evaluated and the diagnosis of recurrence should not be based on an isolated positive PET/CT finding alone. In addition it is mandatory that patients are closely followed and therefore stopping long-term benzimidazole treatment should only be considered in patients who will be compliant with follow-up examinations. In our previous study we could show that especially monitoring with the EmII/3-10 serology is very useful to predict recurrence [18]. Finally as patients safety is crucial, restarting benzimidazole treatment should also be considered in patients requiring any kind of immunosuppressive treatment such as chemotherapy.

## Supporting Information

S1 Checklist CONSORT checklist. (DOC)

S1 Table PNM classification of human alveolar echinococcosis and PNM stage grouping of alveolar echinococcosis. (DOCX)
