# Supplementary material for: Outcome after Discontinuing Long-Term Benzimidazole Treatment in 11 Patients with Non-resectable Alveolar Echinococcosis with Negative FDG-PET/CT and Anti-EmII/3-10 Serology
Source: PLoS Negl Trop Dis. 2015 Sep 21;9(9):e0003964. doi: 10.1371/journal.pntd.0003964 (PMC4577091; doi:10.1371/journal.pntd.0003964)
Supplement: S1 Table — (DOCX) [file pntd.0003964.s002.docx]

**Supporting Information**

**STable: PNM classification of human alveolar echinococcosis and**

| **P** |  | **Hepatic localisation of the Parasite** |
| --- | --- | --- |
|  | **PX** | Primary tumor cannot be assessed |
|  | **P0** | No detectable tumor in the liver |
|  | **P1** | Peripheral lesions without proximal vascular and/or biliary involvement |
|  | **P2** | Central lesions with proximal vascular and/or biliary involvement of one lobe***** |
|  | **P3** | Central lesions with hilar vascular or biliary involvement of both lobes and/ or with Involvement of two hepatic veins |
|  | **P4** | Any liver lesion with extension along the vessels**+** and the biliary tree |
| **N** |  | **Extra hepatic involvement of neighbouring organs** |
|  | **NX** | Not evaluable |
|  | **N0** | No regional involvement |
|  | **N1** | Regional involvement of contiguous organs or tissues**#** |
| **M** |  | **The absence or presence of distant Metastasis** |
|  | **MX** | Not completely evaluated |
|  | **M0** | No metastasis**†** |
|  | **M1** | Metastasis |

***** For classification, the plane projecting between the bed of the gall bladder and the inferior vena cava divides the liver in two lobes.

**+** Vessels defined as the inferior vena cava, portal vein, hepatic veins and arteries

**†** Chest X-ray and cerebral CT negative.

**#** Diaphragm, lung, pleura, pericardium, heart, gastric and duodenal wall, adrenal glands, peritoneum, retroperitoneum, parietal wall (muscles, skin, bone), pancreas, regional lymph nodes, liver ligaments, kidney

**PNM stage grouping of alveolar echinococcosis**

| **Stage** | **P** | **N** | **M** |
| --- | --- | --- | --- |
| Stage I | P1 | N0 | M0 |
| Stage II | P2 | N0 | M0 |
| Stage IIIa | P3 | N0 | M0 |
| Stage IIIb | P1– 3  P4 | N1  N0 | M0  M0 |
| Stage IV | P4  Any P | N1  Any N and/or | M0  M1 |
